# Teratogenic Effects of Coadministration of Fluoxetine and Olanzapine on Rat Fetuses

**DOI:** 10.1155/2014/132034

**Published:** 2014-01-15

**Authors:** Azam Bakhtiarian, Nasrin Takzare, Mehdi Sheykhi, Narges Sistany, Farahnaz Jazaeri, Mario Giorgi, Vahid Nikoui

**Affiliations:** ^1^Department of Pharmacology, School of Medicine, Tehran University of Medical Sciences, Pour Sina Street, Qods Street, Keshavarz Boulevard, Tehran 1417613151, Iran; ^2^Department of Anatomy, School of Medicine, Tehran University of Medical Sciences, Tehran 1417613146, Iran; ^3^School of Medicine, Tehran University of Medical Sciences, Tehran 1417613110, Iran; ^4^Department of Neurosurgery, Shariati Hospital, Tehran University of Medical Sciences, Tehran, Iran; ^5^Department of Veterinary Sciences, University of Pisa, San Piero a Grado, Pisa 56122, Italy

## Abstract

*Objective*. Depression during pregnancy is a relatively common problem. Since little is known about the teratogenic effects of concomitant administration of fluoxetine and olanzapine during the organogenesis period, the aim of the present study was to evaluate the teratogenic effects of coadministration of fluoxetine and olanzapine on rat fetuses. *Method*. Forty-two pregnant rats were divided into seven groups, randomly. The first group received 0.5 mL of normal saline as the control. The second and third groups received fluoxetine at doses of 9 mg/kg and 18 mg/kg, respectively. Olanzapine was injected at 3 mg/kg and 6 mg/kg to the fourth and fifth groups, respectively. The sixth group received 9 mg/kg fluoxetine and 3 mg/kg olanzapine. Finally, the seventh group was administrated with fluoxetine and olanzapine at 18 mg/kg and 6 mg/kg, respectively. Drugs were injected intraperitoneally between day eight and day 15 of the pregnancy. On the 17th day of pregnancy, the fetuses were removed and micro-/macroscopically studied. *Results*. Fetuses of rats receiving high doses of these drugs showed a significant rate of cleft palate development, premature eyelid opening and torsion anomalies, compared to the control group (*P* ≤ 0.01). It is concluded that these drugs can lead to teratogenicity, so their concomitant use during pregnancy should be avoided, or if necessary their doses must be decreased.

## 1. Introduction

One of the most used antidepressant drugs is fluoxetine. This active ingredient belongs to the SSRIs (selective serotonin reuptake inhibitors) class. It increases serotonin levels in synaptic clefts and is used for treatment of depression [1]. It is also used for obsessive-compulsive disorder (OCD), a prevalent disease of enhanced anxiety that has been diagnosed in around 2% of world population. Estrogen and progesterone imbalance and its influence on cerebrospinal fluid partly explain the incidence of psychological problems including OCD during pregnancy [2, 3]. Researchers have shown that OCD can be triggered during fertility periods like menstruation, pregnancy, or postparturition times. Its rate can be decreased by early diagnosis and appropriate treatment [4]. Maina and colleagues have demonstrated the precipitating effect that pregnancy and parturition can have for OCD which leads to postparturition problems for both mother and baby [5]. Leckman and colleagues found that oxytocin secretion during pregnancy increases intracerebral pressure (ICP) and can also lead to OCD [6].

Olanzapine is an atypical antipsychotic drug for treating schizophrenia and other manic syndromes. In 2013, Dubovsky reported that coadministration of fluoxetine and olanzapine has a potentiating impact on the treatment of depression due to their synergic effects [7]. The explanation for this phenomenon is that olanzapine stabilises serotonin levels already increased by fluoxetine treatment. Coadministration of olanzapine and fluoxetine shows synergistic effects on intracellular survival pathways that allow for persistence of the molecules [8]. Administration of prescription drugs during pregnancy should be avoided so as not to detrimentally effect fetal development; however sometimes this is not possible. Following the thalidomide disaster, there has been a significant increase in attention paid to teratogenic properties of drugs during pregnancy. Although drug prescription in pregnancy has decreased, some medications still may be used in pregnant women and could result in problems in development of the fetus [9, 10].

In some cases, pregnant mothers are not aware of their pregnancy in the early months, if they have been prescribed fluoxetine and olanzapine, they would be taking it, oblivious to the possible dangers. The aim of this study is to investigate the teratogenic effects of administration of either olanzapine or fluoxetine or their use in combination at different doses on fetal development in pregnant rats.

## 2. Materials and Methods

### 2.1. Animals

Healthy adult female and male NMRI (Naval Medical Research Institute) rats with an average age of approximately three months and weighing 250–300 grams were randomly selected. They were kept at a temperature of 22 ± 2°C and humidity of 70% and exposed to 12 hours of daylight per day with *ad libitum* access to food and water. After mating and ensuring successful conception, forty-two pregnant rats were randomly divided into seven groups (*n* = 6). All experiments were conducted in Tehran University of Medical Sciences according to the recommendations of the Ethics Committee on Animal Experimentation of the Medical School.

### 2.2. Drugs

Drugs were purchased from Sigma-aldrich Company, USA. Predetermined doses of the drugs were injected intraperitoneally daily, between the eighth and fifteenth day of pregnancy. The first group received 0.5 mL of normal saline as the control. The second and third groups received fluoxetine at doses of 9 mg/kg and 18 mg/kg, respectively. Olanzapine was injected at 3 mg/kg and 6 mg/kg to the fourth and fifth groups, respectively. The sixth group received 9 mg/kg fluoxetine and 3 mg/kg olanzapine. Finally, the seventh group was administrated with fluoxetine and olanzapine at 18 mg/kg and 6 mg/kg, respectively. On the 17th day of pregnancy, the animals were euthanized by inhalation of CO_2_ and the fetuses were removed by caesarean section.

### 2.3. Macroscopic and Microscopic Studies

The fetuses were examined for macroscopic abnormalities. Histopathological slides from fetuses were also prepared. After hematoxylin and eosin staining, any microscopic changes in fetuses were noted using an optical microscope. Positional anomalies (abnormal body shape or non-C-shaped), limb abnormalities (bent limbs), and structural defects (unilateral or bilateral cleft palates and nonfused eyelids) were considered as abnormal fetuses [11].

### 2.4. Statistical Analysis

Data were analysed using statistical software GraphPad Prism version 5. Fisher's exact test was used to ascertain the significance of variations between the numbers of abnormal fetuses in different groups. Differences were considered significant at *P* ≤ 0.01.

## 3. Results

**Figure 1:**
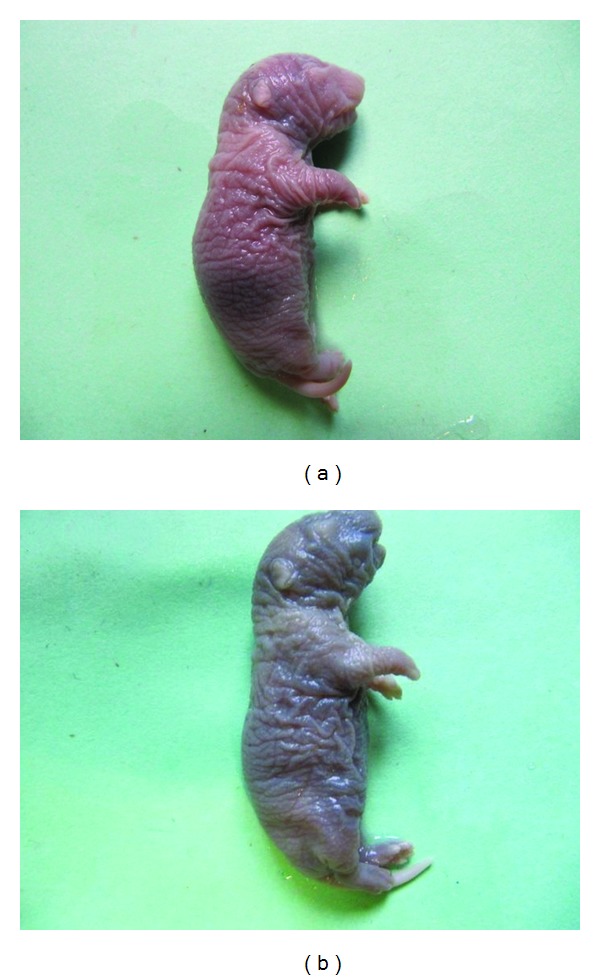
Macroscopic view of 17 days fetuses of control (a) and the group received high doses of fluoxetine and olanzapine (b). In the control group fetus, the body is C-shaped and upper and lower extremities are in their normal locations (a). In the fetus from the group that received high doses of fluoxetine and olanzapine, the body is not fully C-shaped and the forelimbs are not symmetrical (b).

**Figure 2:**
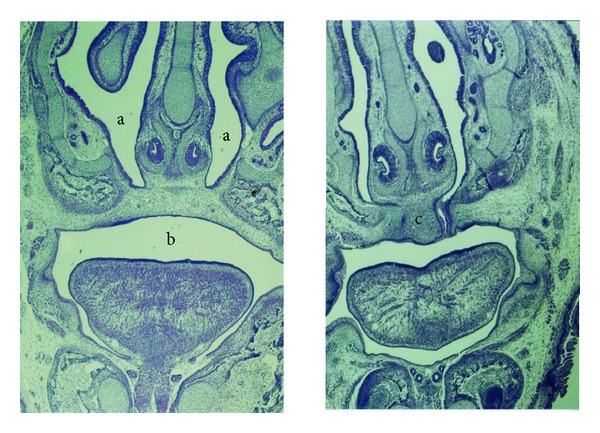
Histopathological slides of the frontal section of the head, 17-day fetuses in the control (left) and the group received high doses of fluoxetine and olanzapine (right) (H&E staining, 4x). In the control group (left), the nasal septum is attached to the roof of the mouth and nostrils (a) are completely separated from the oral cavity (b). In the fetus from the group that received high doses of fluoxetine and olanzapine (right), there is unilateral clefting of the palate (c).

**Figure 3:**
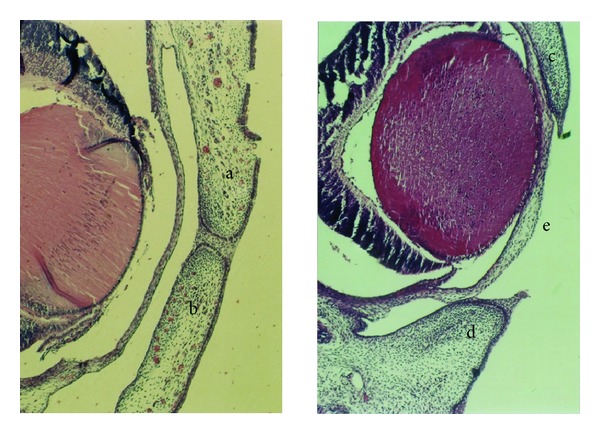
Histopathological slides of the eyes from 17 days fetuses in control (left) and the group received high doses of fluoxetine and olanzapine (right) (H&E staining, 10x). In control group fetus (left), the upper (a) and lower (b) eyelids are joined completely. In the fetus from the group that received high doses of fluoxetine and olanzapine (right), the upper (c) and lower (d) eyelids were separated completely and the cornea (e) is exposed.

No abnormal limbs were noted in the control group; however, in the other groups, multiple fetuses had obvious abnormalities of their limbs and body. Abnormalities in other tissues such as the ear, neck, and tail were not observed in the control, low dose (9 mg/kg) fluoxetine, and high dose (18 mg/kg) fluoxetine groups; however, in the groups treated with low dose (3 mg/kg) olanzapine, high dose (6 mg/kg) olanzapine, combination of fluoxetine and olanzapine in low doses, and combination of fluoxetine and olanzapine in high doses groups, several anomalies were seen. The number of total and abnormal fetuses and litters in different groups are shown in Table 1. Fisher's exact statistical analysis showed that the differences in the number of apparent anomalies between the control group and those that received low dose of olanzapine (3 mg/kg), high dose of olanzapine (6 mg/kg), and combination of fluoxetine and olanzapine in low and high doses are significant (*P* ≤ 0.01). We also found a significant difference in the number of apparent anomalies between groups injected with low and high doses of olanzapine as compared to those treated with a combination of fluoxetine and olanzapine in low and high doses (*P* ≤ 0.01). In the morphological exam, 17-day-old fetuses of the control group had formed their normal C-shaped body with normal extremities. (Figure 1(a)). In the group receiving the highest doses of fluoxetine and olanzapine, fetuses had an abnormal body shape and short limbs (Figure 1(b)). Histopathological slides from frontal sections of control group fetal heads showed that the wall of the nose (nasal septum) was located in the middle of the nasal cavity and was connected to the roof of the mouth. The oral cavity was completely isolated from the nasal cavity (Figure 2(a)). Eyelids were fused together and the cellular layers of eyeball were normal (Figure 3(a)). Microscopic slides of frontal skull sections in the groups receiving the high dose of fluoxetine and a combination of fluoxetine and olanzapine revealed unilateral cleft palates in some samples (Figure 2(b)). In addition, eyelids were not fused together in these groups (Figure 3(b)).

## 4. Discussion

After the thalidomide tragedy, scientists became aware of the importance of considering the teratogenic effects of drugs administered during pregnancy [10]. Drugs with low teratogenicity have no impact on most pregnant women but can be harmful in some cases. The rate of depression increases during pregnancy, and fluoxetine use may be considered.

In the present experiment, we used fluoxetine at doses of 9 mg/kg and 18 mg/kg and olanzapine at doses of 3 mg/kg and 6 mg/kg. Pohland et al. reported that fluoxetine at a dose of 12.5 mg/kg could pass through the placenta and distribute within the fetus during periods of organogenesis in rats [11]. Vorhees et al. have shown that administering fluoxetine at dose of 12 mg/kg caused maternal weight loss during pregnancy, reduced litter sizes at birth, and increased neonatal mortality [12]. Cabrera-Vera et al. showed that prenatal exposure to fluoxetine (10 mg/kg) could produce limited changes in brain serotoninergic neurons in rats [13]. Conversely, Byrd and Markham did not find any teratogenicity in fetuses of rats who had been given fluoxetine at doses of 12.5 mg/kg and lower [14]. In our experiment, we chose the doses of 9 and 18 mg/kg for fluoxetine, based on the above studies.

In 2002, Rosengarten and Quartermain administrated olanzapine at a therapeutic dose of 2 mg/kg/day to pregnant rats [15]. In 1998, Li et al. demonstrated that olanzapine at doses of 0.5, 3, and 10 mg/kg administered subcutaneously exerted pharmacological effects through elevation of extracellular dopamine and norepinephrine levels in rat brains [16]. Aravagiri et al. in 1999 studied the pharmacokinetics and tissue distribution of olanzapine in rats. They performed their experiment with the standard doses of 0.25, 1, 3, and 6 mg/kg/day intraperitoneally [17]. The doses of 3 and 6 mg/kg of olanzapine in this study were chosen based on previous experiments, and these doses caused some anomalies in rat fetuses. Therapeutic doses of fluoxetine and olanzapine for human use are 0.25 to 1 mg/kg/day and 0.1 to 0.25 mg/kg/day, respectively. Since the metabolism of rats is much more efficient than that of humans, we used higher doses than would be used in humans.

Goldstein et al. in 1997 and Oberlander et al. in 2004 reported that most selective serotonin reuptake inhibitors, especially medical doses of fluoxetine, have no teratogenic effects [18, 19]. Many clinical assays have shown similar results [20, 21]. We also found no specific effect of fluoxetine in the present study.

Moses-Kolko et al. in 2005 published a literature review on the fetotoxic effects of some SSRIs [22]. Casper et al. in 2003, Cissoko and colleagues in 2005, and Gentile in 2005 have also mentioned these teratogenic effects [23–25]. Some assays reported that high doses of SSRIs like fluoxetine have potential harmful effects on fetus maturation, but they did not provide sufficient information about the onset and duration of these adverse effects [26–29]. Research has indicated that coadministration of fluoxetine and olanzapine is useful for treating resistant depression [30]. The long half-life of fluoxetine and its active metabolite (dimethyl fluoxetine) increases the chance of drug interaction even after treatment is discontinued [31–33].

Metabolism of the drugs is catalysed by selective cytochrome P_450_ (CYP) isoenzymes. Fluoxetine and its metabolite norfluoxetine are potent inhibitors of the cytochrome CYP2D6 pathway [34], and olanzapine metabolism is dependent on this path [35], so it is possible that fluoxetine increases olanzapine persistence. Drug interactions, especially those involving metabolism, should be considered as an important matter. We have previously shown that coadministration of caffeine and clomipramine during pregnancy could potentiate the teratogenic effects of caffeine in rats [36]. This finding can be attributed to the inhibitory influence of clomipramine on caffeine metabolism via CYP1A2 [37]. It has been established that benzodiazepines, including alprazolam, also cause teratogenic effects, and drug interactions can be involved in this phenomenon too [38]. We observed that groups coadministered with fluoxetine and olanzapine showed more teratogenic features in comparison to groups were administered by fluoxetine alone, and abnormal limb rotations were much more frequent in these combined therapy groups (Table 1). Fluoxetine and olanzapine are categorised in group C of pregnancy medications [39, 40]. So maybe the benefits of using these drugs during pregnancy outweigh their possible risks. Anyway, cautions in their concomitant use during pregnancy should be considered.

## 5. Study Limitations

Since the individual pups are not independent, but belong to litters, the problem of dependence within litters can be considered as a limitation of the present study. Future studies with larger sample sizes using a hierarchic analysis of between-litter and within-litter variances could result in more reliable outcome. Metabolism differences between rats and humans and consequence dosage dissimilarities among these species are also a restriction of this experiment.

## 6. Conclusions

The present research has been carried out in rats, so caution should be used in extrapolating this data to human beings. With this caveat, it might be concluded that because of the potential teratogenic effects of fluoxetine and olanzapine and also the inhibitory effects of fluoxetine on metabolism and elimination of olanzapine, coadministration of these drugs during pregnancy should be avoided, or if necessary their doses must be decreased.

## Figures and Tables

**Table 1:** The number of total and abnormal fetuses and litters in the different groups (F: fluoxetine with doses of 9 and 18 mg/kg and O: olanzapine with doses of 3 and 6 mg/kg).

Groups	Anomalies						
	Total fetuses	Abnormal fetuses	Litters with at least one abnormal fetus	Bent limbs	Non-C-shaped body	Cleft palate	Nonfused eyelids
Control	58	1	1	0	1	0	0
F9	56	2	1	0	0	0	2
F18	59	4	3	4	0	1	4
O3	58	6*	2	3	1	0	2
O6	54	16*	6	7	4	0	7
F9 + O3	57	40^∗†^	6	17	14	4	11
F18 + O6	60	48^∗†^	6	15	17	9	20

Fisher's exact test. *Significant with abnormal fetuses of control group (*P* ≤ 0.01). ^†^Significant with abnormal fetuses of O3 and O6 groups (*P* ≤ 0.01).
